# Machine Learning–Based Prediction of Histopathological Classification in Colorectal Polyps

**DOI:** 10.5152/tjg.2025.25542

**Published:** 2025-10-01

**Authors:** Gökhan Koker, Gizem Zorlu Gorgulugil, Muhammed Ali Coskuner, Merve Eren Durmus

**Affiliations:** 1 Department of Internal Medicine, University of Health Sciences, Antalya Training and Research Hospital, Antalya, Türkiye; 2 Department of Internal Medicine, Antalya City Hospital, Antalya, Türkiye; 3 Department of Gastroenterology, University of Health Sciences, Antalya Training and Research Hospital, Antalya, Türkiye

**Keywords:** Adenomatous polyps, colorectal polyps, diet, histopathology, hyperplastic polyps, machine learning

## Abstract

**Background/Aims:**

Colorectal polyps are precursor lesions of colorectal cancer, and their histopathological types are critical for determining malignant potential. Predicting polyp histopathological types may support early and appropriate clinical management. Machine learning (ML) algorithms based on accessible demographic, clinical, and lifestyle data can contribute to individualized screening strategies.

**Materials and Methods:**

This retrospective cross-sectional study included 491 individuals who underwent colonoscopy for the first time between 2022 and 2025 at University of Health Sciences, Antalya Training and Research Hospital. Demographic and clinical data were recorded, and dietary habits were assessed using the Food Frequency Questionnaire. Patients were classified into 3 groups according to histopathology: adenomatous polyp, hyperplastic polyp, and no polyp. Four ML algorithms—decision tree, random forest, support vector machines (SVMs), and extreme gradient boosting—were applied. Model performance was evaluated using accuracy, sensitivity, specificity, kappa statistic, and McNemar’s test. Variable contributions were further analyzed with SHapley Additive exPlanations.

**Results:**

Accuracy ranged from 70.9% to 76.4%, with the highest performance from SVM (76.4%) and random forest (75.7%). Extreme gradient boosting showed lower overall accuracy (70.9%) but was the only model that identified hyperplastic polyps. The no polyp group was consistently predicted with high accuracy (sensitivity 85.6%-95.9%). Precision for adenomatous polyps was highest with SVM (71.4%). SHapley Additive exPlanations analysis highlighted frequent bulgur consumption (>2 times/week), red meat intake, age, and body mass index as major predictors.

**Conclusion:**

Machine learning algorithms can predict colorectal polyp histopathological types using routine demographic, clinical, and dietary data, enabling more personalized and effective screening beyond age-based protocols.

**Main Points**

- Machine learning algorithms using demographic, clinical, and dietary data can predict histopathological subtypes of colorectal polyps.
- Support vector machine and random forest models achieved the highest accuracy, while extreme gradient boosting was the only model able to classify hyperplastic polyps.
- Frequent bulgur and red meat consumption, age, and body mass index were identified as major predictors for adenomatous polyps.
- These findings highlight the potential of artificial intelligence–based approaches to complement colonoscopy and guide personalized colorectal cancer screening strategies.

## Introduction

Colorectal cancer (CRC) is a leading cause of cancer-related morbidity and mortality worldwide, most often arising from precursor lesions known as colorectal polyps.^1^^,^^2^ Histopathologically, polyps are mainly classified as adenomatous or serrated, the latter including hyperplastic polyps and serrated adenomas. While adenomas follow the adenoma–carcinoma sequence, serrated lesions account for 15%-30% of CRC through the serrated pathway.^3^ Screening colonoscopy and timely removal of these lesions significantly reduce CRC incidence and mortality.^2^

Diet and lifestyle factors play an important role in both CRC and polyp development. Smoking, alcohol, high red and processed meat intake, and inactivity increase polyp risk, whereas fiber-rich diets and certain chemopreventive agents are protective.^3^^,^^4^ Recent evidence also suggests etiologic heterogeneity: while obesity and smoking affect both adenomas and serrated lesions, whole-grain intake appears protective mainly for adenomas, and high white meat intake is more strongly linked to serrated polyps.^3^ Such findings indicate that lifestyle factors may influence not only the occurrence but also the histopathological type of polyps.

The histologic subtype of a polyp has direct clinical implications. Adenomas, particularly those with advanced features, carry significant malignant potential, while most small hyperplastic polyps in the rectosigmoid colon pose minimal risk.^5^ Accordingly, surveillance guidelines stratify follow-up based on polyp type, size, and number.^2^ Accurate histological prediction could therefore guide management decisions, including “resect-and-discard” or “leave-in-situ” strategies for diminutive polyps.^6^ However, optical diagnosis techniques such as narrow-band imaging often show variable accuracy, particularly among non-experts.^1^ Misclassification risks either unnecessary interventions or missed neoplastic lesions, underscoring the need for reliable, objective tools.

Recent advances in artificial intelligence and machine learning (ML) provide promising approaches for real-time polyp characterization. Deep learning algorithms have achieved accuracies above 90% in differentiating adenomatous from hyperplastic polyps, even in diminutive lesions.^1^ Radiomics-based ML applied to computed tomography colonography also showed high accuracy in distinguishing adenomatous from non-adenomatous polyps.^2^ A systematic review confirmed convolutional neural networks have high sensitivity and specificity for polyp and cancer diagnosis.^7^ These findings highlight the potential of ML to enhance histopathological prediction, optimize surveillance strategies, and improve patient care.^1^^,^^6^

## Materials and Methods

This retrospective cross-sectional study was conducted at the Department of Gastroenterology, University of Health Sciences, Antalya Training and Research Hospital, between January 2022 and April 2025. A total of 491 individuals who underwent colonoscopy for the first time were included, while patients with a history of colorectal cancer, previous colorectal surgery, or prior detection of polyps were excluded. Written informed consent was obtained from all participants who agreed to take part in the study. Demographic and clinical variables, including age, sex, body mass index (BMI), education level, family history of colorectal polyps, history of constipation, nonsteroidal anti-inflammatory drug (NSAID), aspirin or vitamin supplement use, alcohol consumption, tobacco use, and exercise habits, were recorded. Dietary habits were assessed using the validated Food Frequency Questionnaire, which provided information on the frequency of consumption of red meat, white meat, fish, salad, cooked vegetables, tomatoes, legumes, nuts, fruits, bulgur, rice, bread, cheese, milk, and eggs; dietary intake was categorized as “never,” “1-3 times per month,” “1-2 times per week,” “4-7 times per week,” or “daily.” Histopathological examination results classified patients into 3 groups: adenomatous polyp, hyperplastic polyp, and no polyp (normal mucosa). To predict polyp histopathological types, 4 ML algorithms—decision tree, random forest, support vector machines (SVMs), and extreme gradient boosting (XGBoost)—were applied. The dataset was randomly divided into a training set (70%) and a test set (30%), and model performance was evaluated using accuracy, sensitivity, specificity, positive and negative predictive values, F1-score, kappa statistic, and McNemar’s test. To identify influential predictors, recursive feature elimination was applied, and SHapley Additive exPlanations (SHAP) analysis was performed to assess the contribution of each variable to model classification decisions. All analyses were conducted using the R programming language (version 4.4.3) (R Foundation for Statistical Computing; Vienna, Austria). For all statistical tests, including McNemar’s test, a *P*-value <.05 was considered statistically significant. Ethical approval for this study was obtained from the University of Health Sciences Antalya Training and Research Hospital Ethics Committee (Decision No.: 7/7; Date: April 17, 2025).Written informed consent was obtained from all participants included in the study.

## Results

A total of 491 individuals were included in the study. The mean age was 52.0 ± 13.5 years (range 18-86), with 283 (57.6%) females and 208 (42.4%) males. The mean BMI was 26.95 ± 5.4 kg/m2 (range 15.4-47.1). A family history of colorectal polyps was present in 13.4% (n = 66) of participants. Regarding educational level, 11.2% (n = 55) were illiterate, 68.4% (n = 336) had primary or secondary education, and 20.4% (n = 100) were university graduates. With regard to lifestyle habits, 38.3% (n = 188) reported no exercise, 54.8% (n = 269) exercised occasionally, and 6.9% (n = 34) exercised regularly. Alcohol and tobacco use were reported by 9.4% (n = 46) and 18.7% (n = 92), respectively. Constipation was present in 40.9% (n = 201), NSAID use in 45.0% (n = 221), aspirin use in 20.2% (n = 99), and vitamin supplementation in 27.5% (n = 135). Detailed baseline characteristics are presented in Table 1.

Histopathological examination revealed adenomatous polyps in 24.8% (n = 122), hyperplastic polyps in 9.3% (n = 46), and no polyps in 65.9% (n = 323). This class imbalance, particularly the low frequency of hyperplastic polyps, was expected to affect model performance.

**Figure 1.**
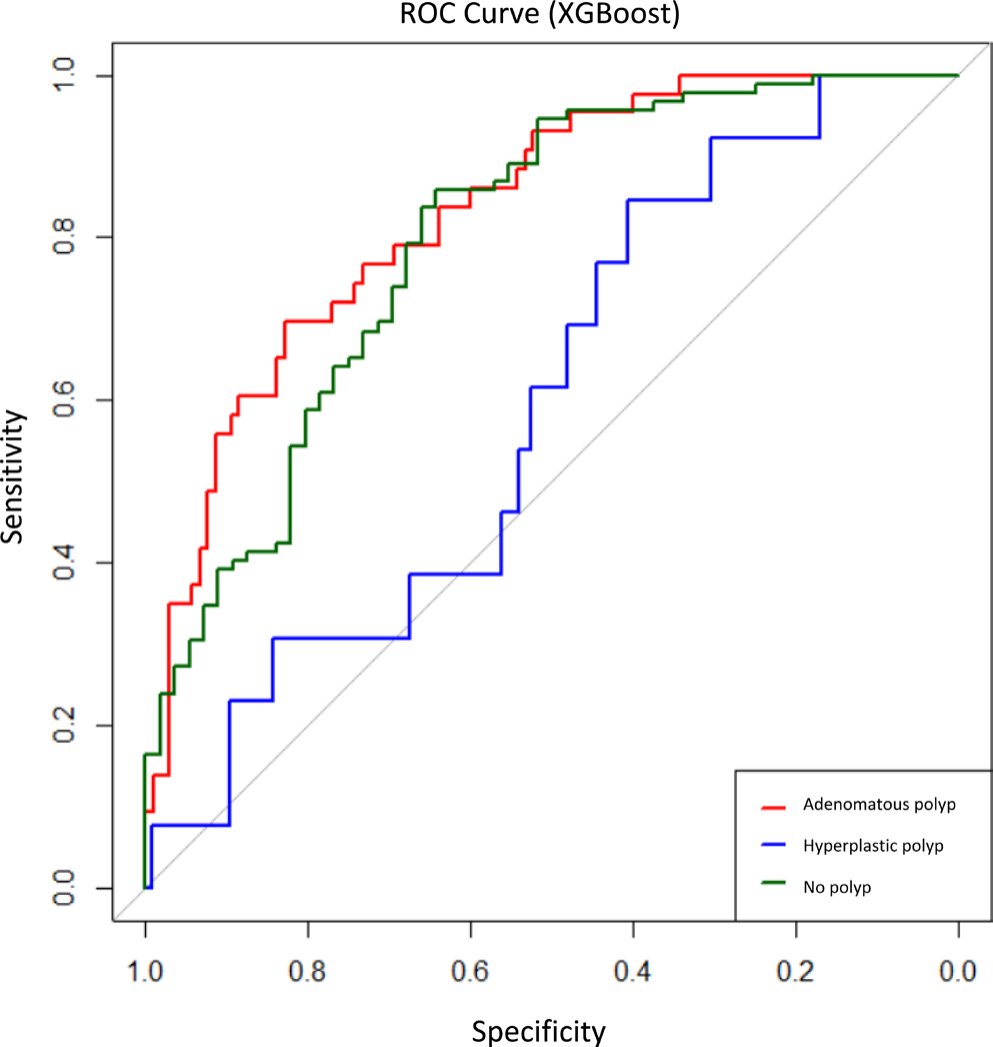
Receiver operating characteristic curves generated by the extreme gradient boosting algorithm, demonstrating the sensitivity and specificity performance of the model for each pathological class.

**Figure 2.**
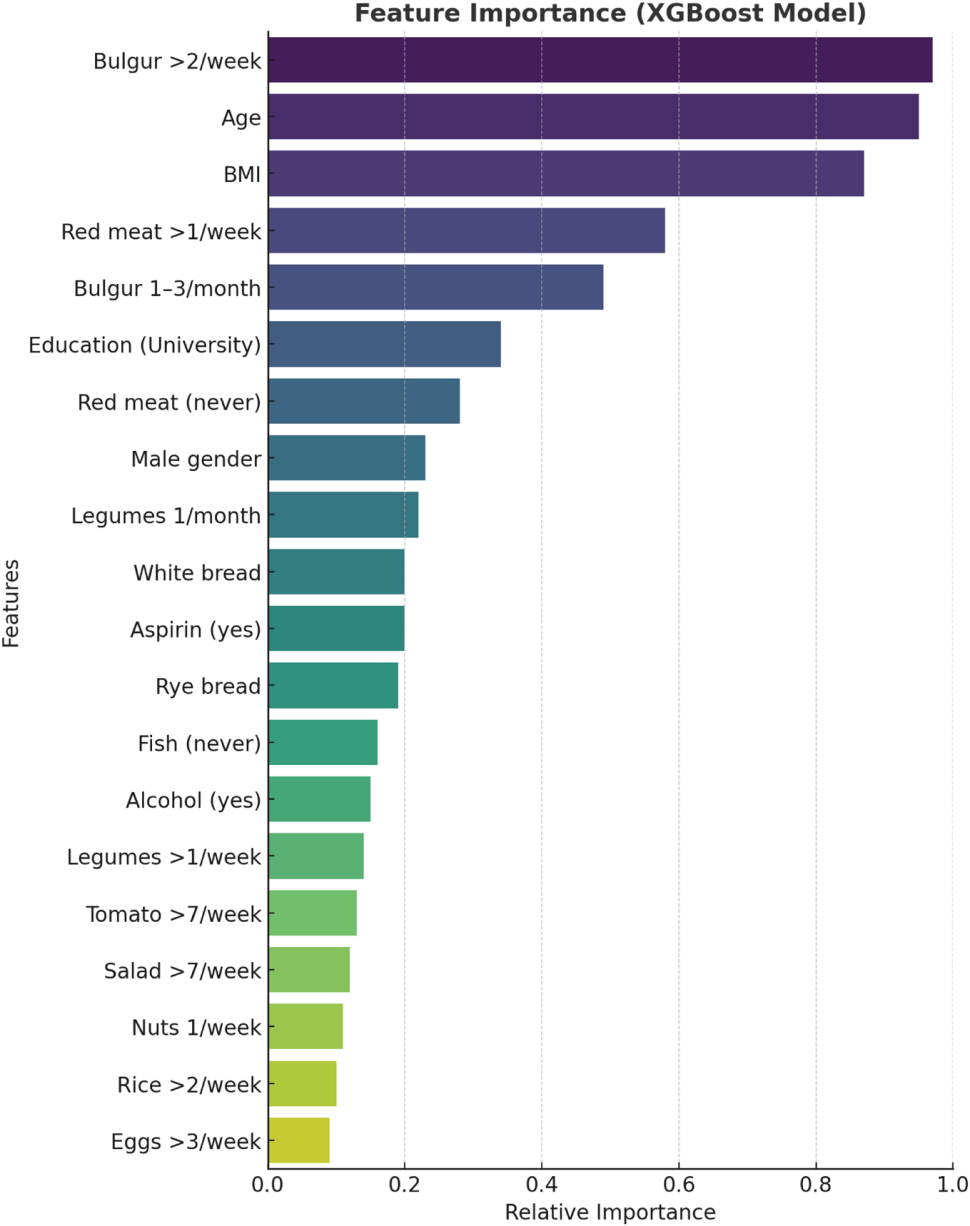
Feature importance plot generated for the extreme gradient boosting model.

The performance of 4 ML algorithms—decision tree, random forest, SVMs, and XGBoost—was comparatively evaluated (Table 2). The highest classification accuracies were achieved with SVM (76.4%) and random forest (75.7%), with kappa coefficients of 0.44 and 0.43, respectively, indicating moderate discriminatory ability across the 3 histopathological classes. The decision tree model achieved an accuracy of 73.6% and a kappa of 0.39 but failed to classify hyperplastic polyps, reflecting its susceptibility to class imbalance. In contrast, the XGBoost algorithm yielded a lower overall accuracy (70.9%) but was the only model capable of partially identifying hyperplastic polyps (sensitivity 21.4%, precision 25%). Receiver operating characteristic curves for the XGBoost model demonstrated higher discriminative performance for adenomatous and no polyp classes compared to hyperplastic polyps (Figure 1). Feature importance analysis identified bulgur consumption >2 times/week, age, and BMI as the most influential predictors (Figure 2).

Across all models, the no polyp class was consistently predicted with the highest accuracy (sensitivity 85.6%-95.9%), while precision for adenomas was highest with the SVM model (71.4%). Hyperplastic polyps remained the most difficult group to classify, likely due to their limited representation. McNemar’s test indicated systematic misclassifications for decision tree, random forest, and SVM models, whereas XGBoost provided a more balanced distribution of predictions (*P* = .308).

**Figure 3.**
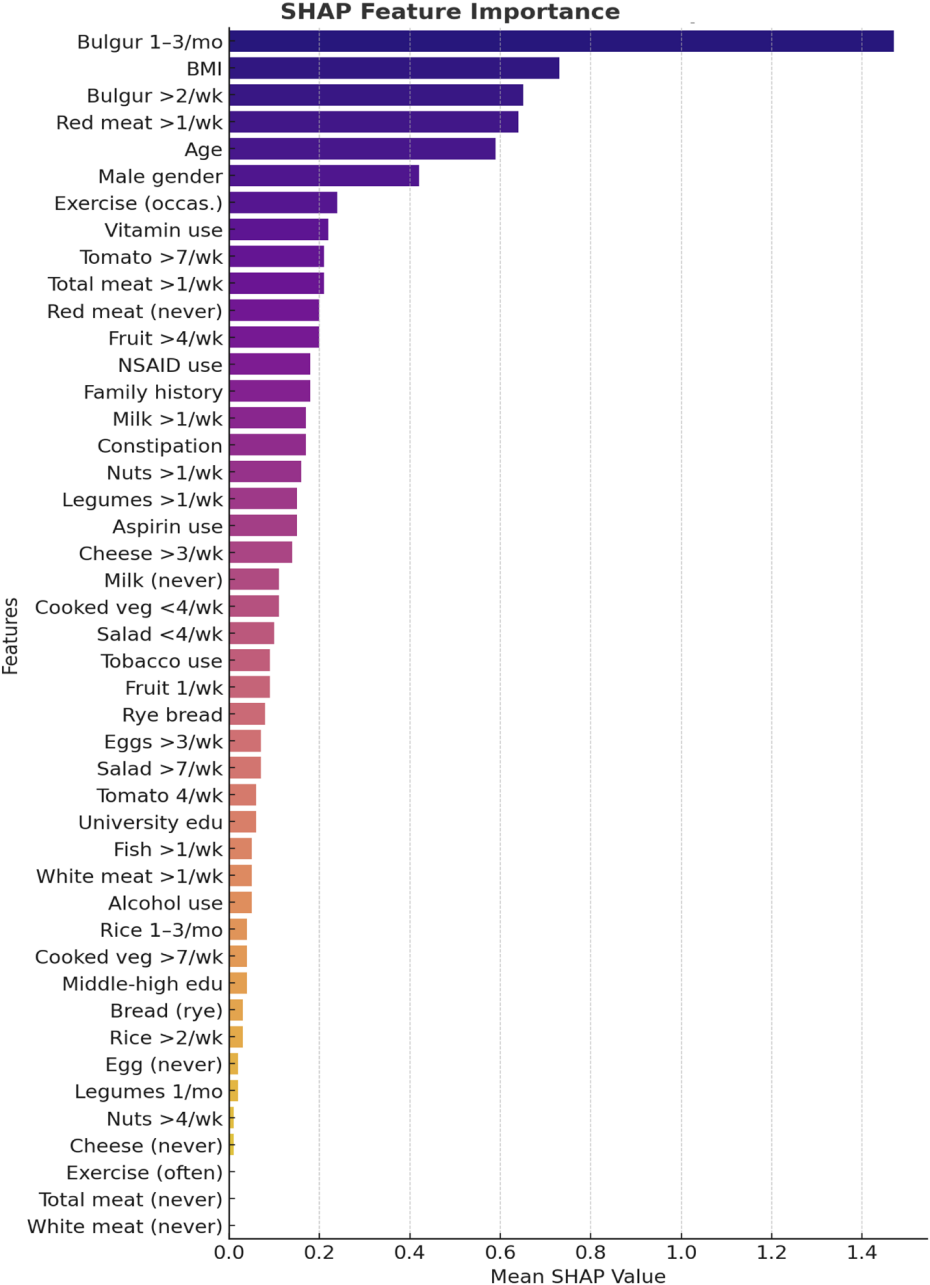
SHapley Additive exPlanations summary plot.

Model interpretability analyses further highlighted the impact of diet and lifestyle variables. SHapley Additive exPlanations values demonstrated that frequent bulgur consumption and higher red meat intake significantly increased the likelihood of adenomatous polyps, while BMI and age were also among the strongest predictors (Figure 3). The decision tree analysis indicated that dietary patterns were sequentially associated with adenoma risk. Individuals consuming bulgur more than twice per week demonstrated an increased probability of adenoma, which was further elevated when red meat consumption exceeded once per week.

Overall, the findings demonstrate that colorectal polyp histopathological subtypes can be predicted using readily accessible demographic, lifestyle, and dietary data. While XGBoost showed unique strength in identifying hyperplastic polyps, class imbalance remains a major limitation. Expanding sample size and applying class-balancing techniques may improve future model performance, particularly for underrepresented subtypes.

## Discussion

The rapid expansion of digital health applications and artificial intelligence (AI)-based technologies has underscored the increasing importance of personalized approaches in medicine. Artificial intelligence algorithms are now widely employed across healthcare, from patient monitoring to diagnosis, treatment planning, and risk prediction, becoming robust tools that support clinical decision-making. In this context, advanced analytical methods are gaining traction in stratifying colorectal polyp risk and aiding histopathological classification.

The most common AI applications in colorectal polyp classification involve the use of deep learning on endoscopic images, with impressive diagnostic accuracy. Shen et al^8^ developed an EfficientNet-b0–based model using approximately 256 000 colonoscopy images, achieving >97% sensitivity and specificity, with an area under the curve (AUC) of 0.9989 for polyp classification in the test set. Similarly, Krenzer et al^9^ reported that AI-assisted systems based on NICE and Paris classifications could differentiate polyp subtypes with accuracies of ~89% and ~81%. Beyond image-based approaches, recent work has highlighted the potential of demographic, clinical, and dietary data as predictors in ML models. For example, Hussan et al^10^ constructed ML models to predict colorectal neoplasia and high-risk polyps in individuals aged 35-50 using accessible data from electronic health records, achieving an AUC of 0.75 with neural networks, outperforming logistic regression.

In the present study, 4 ML algorithms were applied—decision tree, random forest, SVM, and XGBoost—to predict colorectal polyp histopathology using demographic, clinical, and dietary features. Support vector machine (76.4%) and random forest (75.7%) achieved the highest accuracies with moderate kappa values (0.44 and 0.43), while the decision tree model was limited by class imbalance, failing to identify hyperplastic polyps. Extreme gradient boosting yielded lower accuracy (70.9%) but was uniquely capable of partially identifying the hyperplastic group, albeit with modest sensitivity (21.4%) and precision (25%).

Comparable findings have been reported in the literature. Ba et al^11^ developed a polyp prediction model using laboratory and demographic data from 5426 patients, achieving the best performance with AdaBoost (AUC = 0.675), although it required advanced laboratory inputs such as carcinoembryonic antigen (CEA) and HbA1c. Li et al^12^ introduced the “Feature Interpretability Screening Framework” to stratify high-risk colorectal cancer patients, reporting the highest sensitivity with Naïve Bayes and SVM (77.9%) and an AUC of 0.859 with logistic regression. Random forest achieved an AUC of 0.826, comparable to the current results, though targeting different lesion groups. Similarly, Huang et al^13^ applied random forest to evaluate the interplay between gut microbiota and KRAS mutations in CRC, achieving an AUC of 0.819. In another study including 164 patients, random forest reached an AUC of 0.820, with BMI, platelet count, hemoglobin, triglycerides, and aspartate aminotransferase (AST) as the top predictors.^14^

Additional evidence highlights the promise of XGBoost. Li et al^15^ demonstrated that XGBoost outperformed FOBT and CEA in differentiating between healthy controls, polyp patients, and CRC, with AUC values of 0.966 and 0.881, respectively. SHapley Additive exPlanation analysis identified fecal occult blood test (FOBT), CEA, lymphocyte percentage, and hematocrit as the most influential features. In this study, XGBoost also emphasized diet and anthropometrics, with frequent bulgur and red meat intake, BMI, and age emerging as key determinants. Shi et al^16^ further validated the use of XGBoost and SHAP in post-polypectomy recurrence prediction, achieving AUCs of 0.909-0.963 across training, validation, and prospective datasets; smoking history, family history, and age were the strongest predictors. These findings align with these results, with SHAP analyses consistently identifying bulgur and red meat intake as strong dietary correlates of adenomas. The decision tree visualization in the current dataset also underscored these features, demonstrating a clear link between frequent bulgur/red meat intake and adenoma classification.

Although image-based AI systems have reported higher diagnostic accuracies (>90%) in differentiating adenomatous from hyperplastic polyps,^17^ the current model is fundamentally different in that it relies solely on demographic and dietary parameters. Previous studies have demonstrated that demographic and lifestyle-related factors, such as age, sex, smoking, BMI, and diet, are independently associated with colorectal adenoma risk.^18^ Such an approach has potential value in several clinical contexts. First, it may serve as a pre-procedural risk stratification tool, allowing clinicians to identify individuals with a higher probability of adenomatous polyps prior to colonoscopy. Second, in settings with limited resources, where advanced endoscopic imaging or AI-based systems are not available, a demographic- and dietary-based model may provide a practical and low-cost adjunct for risk prediction. Finally, this type of model could play a complementary role when combined with endoscopic AI systems, potentially enhancing diagnostic precision by integrating patient-related factors with imaging features. Therefore, despite its lower accuracy compared with imaging-based AI, the proposed model may still contribute to clinical decision-making in selected scenarios.

The main limitation of this study was the imbalance across histopathological classes, particularly the underrepresentation of hyperplastic polyps, which constrained model performance and produced Not a Number (NaN) values for this group in some models. The limited number of hyperplastic polyps might have influenced the classification accuracy for this subgroup, and this should be considered when interpreting the results. Addressing this limitation through larger and more balanced datasets, as well as the application of class-weighting techniques, will be essential in future research.

In summary, this study demonstrates that ML algorithms can predict colorectal polyp histopathological types using accessible demographic, lifestyle, and dietary data. These results highlight the potential of ML to complement endoscopic and histopathological assessment, paving the way for integration into clinical decision support systems. Such models may provide additional decision support for clinicians and contribute to more individualized screening strategies. Future studies with broader and more balanced cohorts may enhance model accuracy and robustness, ultimately facilitating more personalized screening and surveillance strategies.

The present study demonstrates that histopathological subtypes of colorectal polyps can be predicted using readily accessible demographic, lifestyle, and dietary factors. Specific dietary components, such as frequent bulgur and red meat consumption, in combination with age and BMI, emerged as significant predictors of polyp type. These findings highlight the potential for developing individualized screening strategies that extend beyond the conventional age-based recommendations, allowing risk stratification tailored to each patient’s profile.

Machine learning models, particularly advanced algorithms such as XGBoost, not only achieved competitive classification accuracy but also provided interpretability through SHAP analyses, underscoring the contribution of modifiable lifestyle factors to polyp histopathology. By integrating such predictive models into clinical practice, it may be possible to optimize colorectal cancer screening, facilitate early detection of adenomatous polyps, and simultaneously avoid unnecessary interventions for low-risk hyperplastic polyps, thereby enhancing patient safety and resource efficiency.

Nevertheless, the limited sample size of the hyperplastic subgroup represents a key constraint of the present study, potentially restricting model generalizability. Future investigations with larger, more balanced, and preferably multicenter datasets are warranted to confirm these findings. If validated, explainable AI-based tools hold great promise for integration into clinical decision support systems, ultimately contributing to more effective and personalized colorectal cancer prevention strategies.

## Figures and Tables

**Table 1.** Baseline Ddemographic, Clinical, and Histopathological Characteristics of the Study Population

Variable	Value
Age (years), mean ± SD (min-max)	52.0 ± 13.5 (18-86)
Sex, n (%)	
Female	283 (57.6)
Male	208 (42.4)
BMI (kg/m2), mean ± SD (min-max)	26.95 ± 5.4 (15.4-47.1)
Family history of polyps, n (%)	66 (13.4)
Education level, n (%)	
Illiterate	55 (11.2)
Primary/secondary	336 (68.4)
University	100 (20.4)
Exercise habit, n (%)	
None	188 (38.3)
Occasional	269 (54.8)
Regular	34 (6.9)
Alcohol use, n (%)	46 (9.4)
Tobacco use, n (%)	92 (18.7)
Constipation, n (%)	201 (40.9)
NSAID use, n (%)	221 (45.0)
Aspirin use, n (%)	99 (20.2)
Vitamin supplementation, n (%)	135 (27.5)
Histopathology, n (%)	
Adenomatous polyp	122 (24.8)
Hyperplastic polyp	46 (9.3)
No polyp	323 (65.9)

BMI, body mass index; NSAID, nonsteroidal anti-inflammatory drug.

**Table 2.** Comparison of Machine Learning Models for Colorectal Polyp Pathology Classification

Model	Accuracy (%)	Kappa	Adenoma (Sens/Prec)	Hyperplastic (Sens/Prec)	No Polyp (Sens/Prec)	McNemar *P*	Notes
Decision tree	73.6	0.39	54.1%/62.5%	0% / N/A	91.8%/78.8%	<.001	Hyperplastic class not predicted at all
Random forest	75.7	0.43	56.8%/67.7%	0% / N/A	93.8%/77.8%	<.001	One of the most balanced and accurate models
SVM	76.4	0.44	54.1%/71.4%	0% / N/A	95.9%/77.5%	<.001	Provided highest precision for adenoma
XGBoost	70.9	0.39	51.3%/61.3%	21.4%/25.0%	85.6%/79.0%	.308	The only model that predicted the hyperplastic class

N/A, not avaible; Prec, precision; Sens, sensitivity; SVM, support vector machine; XGBoost, extreme gradient boosting.
